# Single-cell profiling reveals CCL5^Hi^ GZMA^Hi^ effector memory CD8 T cell association to oligoarticular JIA

**DOI:** 10.1093/rheumatology/keag211

**Published:** 2026-04-27

**Authors:** Mireia Lopez-Corbeto, Yolanda Guillén, Laura Jiménez-Gracia, Estefanía Moreno-Ruzafa, Damiana Alvarez-Errico, Núria Palau, Nieves Martín-Begué, Holger Heyn, Antonio Julià, Sara Marsal

**Affiliations:** Paediatric Rheumatology Unit, Rheumatology Department, Vall d’Hebron Barcelona Hospital Campus, Barcelona, Spain; IMIDomics, Inc, Barcelona, Spain; Centro Nacional de Análisis Genómico, Barcelona, Spain; Universitat de Barcelona (UB), Barcelona, Spain; Paediatric Rheumatology Unit, Rheumatology Department, Vall d’Hebron Barcelona Hospital Campus, Barcelona, Spain; IMIDomics, Inc, Barcelona, Spain; Rheumatology Research Group, Rheumatology Department, Vall Hebron Barcelona Hospital Campus, Barcelona, Spain; Paediatric Ophtalmology Unit, Ophtalmology Department, Vall d’Hebron Barcelona Hospital Campus, Barcelona, Spain; Centro Nacional de Análisis Genómico, Barcelona, Spain; Universitat de Barcelona (UB), Barcelona, Spain; ICREA, Barcelona, Spain; Rheumatology Research Group, Rheumatology Department, Vall Hebron Barcelona Hospital Campus, Barcelona, Spain; Rheumatology Research Group, Rheumatology Department, Vall Hebron Barcelona Hospital Campus, Barcelona, Spain

**Keywords:** JIA, uveitis, single-cell RNA sequencing, TCR sequencing

## Abstract

**Objectives:**

Oligoarticular JIA (oligo JIA) is the most common JIA subtype and is often complicated by uveitis, a potentially sight-threatening comorbidity. Despite its prevalence, the immunopathogenic mechanisms underlying oligo JIA and its extra-articular manifestations remain poorly understood. The objective was to characterize the immune landscape of oligo JIA and identify pathogenic cell populations and regulatory mechanisms associated with the disease and uveitis.

**Methods:**

Single-cell RNA and TCR sequencing (scRNA/TCR-Seq) was performed on peripheral blood mononuclear cells (PBMCs) and paired SF from three treatment-naïve, new-onset oligo JIA patients, on PBMC from four oligo JIA patients with uveitis flare and from four age-matched healthy controls (HCs) (discovery cohort; *n* = 14 samples). Cellular composition, gene expression and T cell clonality were analysed. Key findings were validated by mass cytometry (CyTOF) in an independent cohort of 13 oligo JIA patients, eight uveitis flare patients and six HCs.

**Results:**

scRNA-seq of 132 824 immune cells revealed enrichment of activated effector memory CD8^+^ T cells (CD8^+^TEM), intermediate monocytes and regulatory T cells in SF. CD8^+^TEM were depleted in blood, suggesting a recruitment from the circulation into the inflamed joint. SF CD8^+^TEM displayed upregulation of CCL5, GZMA and GNLY and showed marked clonal expansion. In patients with uveitis, MAIT cells were clonally expanded and transcriptionally reprogrammed with IFN-stimulated and cytotoxic signatures. CyTOF confirmed reduced circulating CCL5^Hi^ GZMA^Hi^ CD8^+^TEM in oligo JIA.

**Conclusions:**

Clonally expanded, cytotoxic CD8^+^ TEM cells drive joint inflammation in oligo JIA, while activated MAIT and NK cells in uveitis indicate systemic immune activation and potential therapeutic targets.

## Graphical abstract

**Graphical Abstract:**
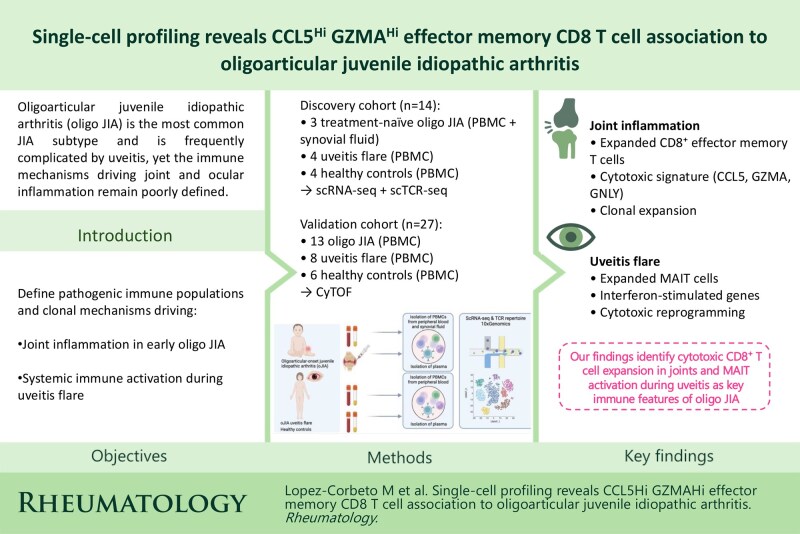

Rheumatology key messages

- Oligoarticular JIA frequently associates with uveitis, but early immune drivers remain poorly defined.
- Single-cell profiling of treatment-naïve disease reveals clonally expanded cytotoxic CD8^+^ T cells, with NK/MAIT activation in uveitis.
- Early systemic immune priming highlights novel therapeutic targets and supports precision, single-cell-guided paediatric rheumatology.

## Introduction

JIA is defined as arthritis persisting for over 6 weeks and starting before 16 years of age. It includes heterogeneous patient groups with distinct trajectories, comorbidities and treatment responses [1]. Oligoarticular JIA (oligo JIA) is the most common subtype, characterized by asymmetric synovial inflammation and an increased risk of uveitis [2].

The pathogenesis of oligo JIA remains unclear but likely involves genetic susceptibility and environmental triggers [3]. Genome-wide association studies have identified major risk variation within the MHC region [4], though subtype-specific mechanisms remain elusive due to small and heterogeneous cohorts. Recent advances in single-cell RNA sequencing (scRNA-Seq) enable high-resolution profiling of immune cells, providing new insights into autoimmune pathogenesis.

Few scRNA-Seq studies have focused on oligo JIA. Analysis of synovial Tregs and CD4+ T cells showed Th1 polarization [5], while broader profiling of paired blood and SF revealed exhausted CD4+ and CD8+ T cell subsets [6]. However, these studies included patients with established disease and adult controls, limiting interpretation.

Here, we present the first scRNA-Seq study of early-onset oligo JIA, comparing patients with age- and sex-matched healthy children. We analysed the full mononuclear cell compartment from paired blood and SF samples, integrated single-cell TCR sequencing to assess clonal dynamics and validated key findings in an independent cohort using mass cytometry (CyTOF).

## Methods

### Study design and study participants

For the scRNA-seq study, three newly diagnosed, treatment-naïve oligo JIA patients (mean age 5 years) were recruited from the Paediatric Rheumatology Unit, Rheumatology Department, Vall d’Hebron Barcelona Hospital Campus. In collaboration with the Paediatric Ophthalmology Department, four oligo JIA patients with uveitis flare were included. Four age- and gender-matched healthy children were recruited as controls (Supplementary Table S1).

For the validation study using mass cytometry, 13 oligo JIA patients, eight patients with uveitis flare and six sex- and age-matched healthy controls (HCs) were recruited (Supplementary Table S2). Blood and SF samples were obtained from new-onset oligo JIA patients prior to any immunomodulatory therapy, including NSAIDs or systemic CSs. Blood samples from uveitis patients were collected at flare detection and from HCs. Patients sampled during uveitis flare had established oligo JIA and were characterized regarding disease duration, arthritis activity at the time of ocular flare and current immunomodulatory treatment.

Written informed consent was obtained for all participants.

The study was approved by the Ethical Committee for Clinical Research of the Vall d’Hebron Barcelona Hospital Campus (PR-AMI-2342018).

### Cell isolation

SF was obtained by arthrocentesis, transferred to heparinized tubes, treated with hyaluronidase (0.2 per 6 ml of SF) during 30 min at 37°C to reduce viscosity, diluted with RPMI and processed within 2 h of collection. SF mononuclear cells (SFMCs) and peripheral blood mononuclear cells (PBMCs) were isolated by Ficoll gradient centrifugation and cryopreserved by controlled-rate freezing, then stored in vapor-phase liquid nitrogen. Although rapid standardized processing was applied to all samples, we acknowledge that short *ex vivo* handling may influence transcriptional profiles.

### Single-cell RNA/TCR sequencing

Cryopreserved PBMCs and SF cells from oligo JIA patients, PBMCs from JIA-associated uveitis patients and matched HCs were thawed and assessed for viability. Live cells were FACS-sorted (FACSAria™ Fusion, BD Biosciences, San Jose, CA and Milpitas, CA).

Cell hashing was performed using TotalSeq™-C antibodies (BioLegend). In paired samples, SF cells were labelled with hashtag 1 and PBMCs with hashtags 2–4; unpaired PBMCs were labelled with hashtags 1–4. Cells were pooled (3:1 for paired samples), filtered and loaded onto the Chromium Controller (10X Genomics; 25 000 cells/sample).

Single-cell encapsulation and library preparation were performed using Chromium Next GEM Single Cell V(D)J v1.1 kits (10X Genomics). Libraries were sequenced on an Illumina NovaSeq 6000 (∼40K read pairs/cell for gene expression; ≥5K for TCR and HTO).

### Single-cell RNA sequencing analytics

Sequencing reads were processed using CellRanger (v5, 10X Genomics) with the GRCh38 reference genome and VDJ database. Cell hashtags were demultiplexed following Stoeckius *et al.* [7] using centred log ratio normalization, log-transformed and processed with HTODemux in Seurat. Multiplets and unassigned barcodes were excluded.

Quality control and normalization were performed following Luecken *et al.* [8], removing low-quality barcodes (<225 UMIs, <100 genes or >20% mitochondrial reads), high-complexity cells (>7000 UMIs or >2000 genes) and genes expressed in <5 cells. Data were normalized and log transformed.

### Single-cell transcriptome combined analysis

Batch effects were corrected using Seurat integration with reciprocal PCA across 11 libraries. UMAP was used for visualization. The first 20 principal components were used to construct a *k*-nearest neighbours graph, followed by Louvain clustering (resolution 0.5). Clusters were annotated using canonical immune markers. Dead cells were excluded, and each lineage was reprocessed with lineage-specific parameters. Cell composition differences were tested using *t*-tests and paired analyses.

### Single-cell TCR repertoire profiling

Clonotypes were defined by donor origin, V(D)J genes and CDR3 annotations for TCR alpha and beta chains. Clonal expansion was assessed using the Gini coefficient across blood and SF samples [9]. Following Yost *et al.*, [10] only T cells with ≥10% TCR sequencing were included. Clonotypes were classified as expanded (≥2 clones) or non-expanded.

### Differential expression analysis

Differential expression was assessed for: (1) SF vs matched peripheral blood in oligo JIA, (2) peripheral blood from oligo JIA patients without uveitis vs HCs and (3) peripheral blood from oligo JIA patients with uveitis vs HCs. Paired analyses used the generalized linear model implemented in MAST, while unpaired analyses used the Wilcoxon signed-rank test. Genes with |log2FC| > 0.25, detected in ≥10% of cells, and adjusted *P* < 0.001 were considered significant. Functional enrichment was performed using Gene Ontology analysis (GO_Biological_Processes_2023) via the GSEA package [11].

### CyTOF staining and analysis

Cryopreserved samples were thawed, rested overnight and processed using standard Maxpar protocols. Cells were stained with a 31-antibody panel, fixed, barcoded with Cell-ID Intercalator-Ir and acquired on a Helios™ mass cytometer using EQ calibration beads.

CyTOF data analysis combined unsupervised and supervised approaches, using optSNE for dimensionality reduction and FlowSOM for clustering. Analyses were conducted using OMIQ, R and Python.

### Code and data availability

Analyses were performed in R and Python using Seurat, Scirpy and scRepertoire [12]. Sequencing data have been deposited in the NCBI repository. Given the exploratory nature and limited sample size of the discovery cohort, a nominal threshold of *P* < 0.1 was used to identify candidate differences in cellular abundance. Exact *P*-values are provided in Supplementary materials. Additional data are available from the corresponding author upon reasonable request.

## Results

### Cellular landscape of oligo JIA, oligo JIA uveitis flare and HCs

**Figure 1:**
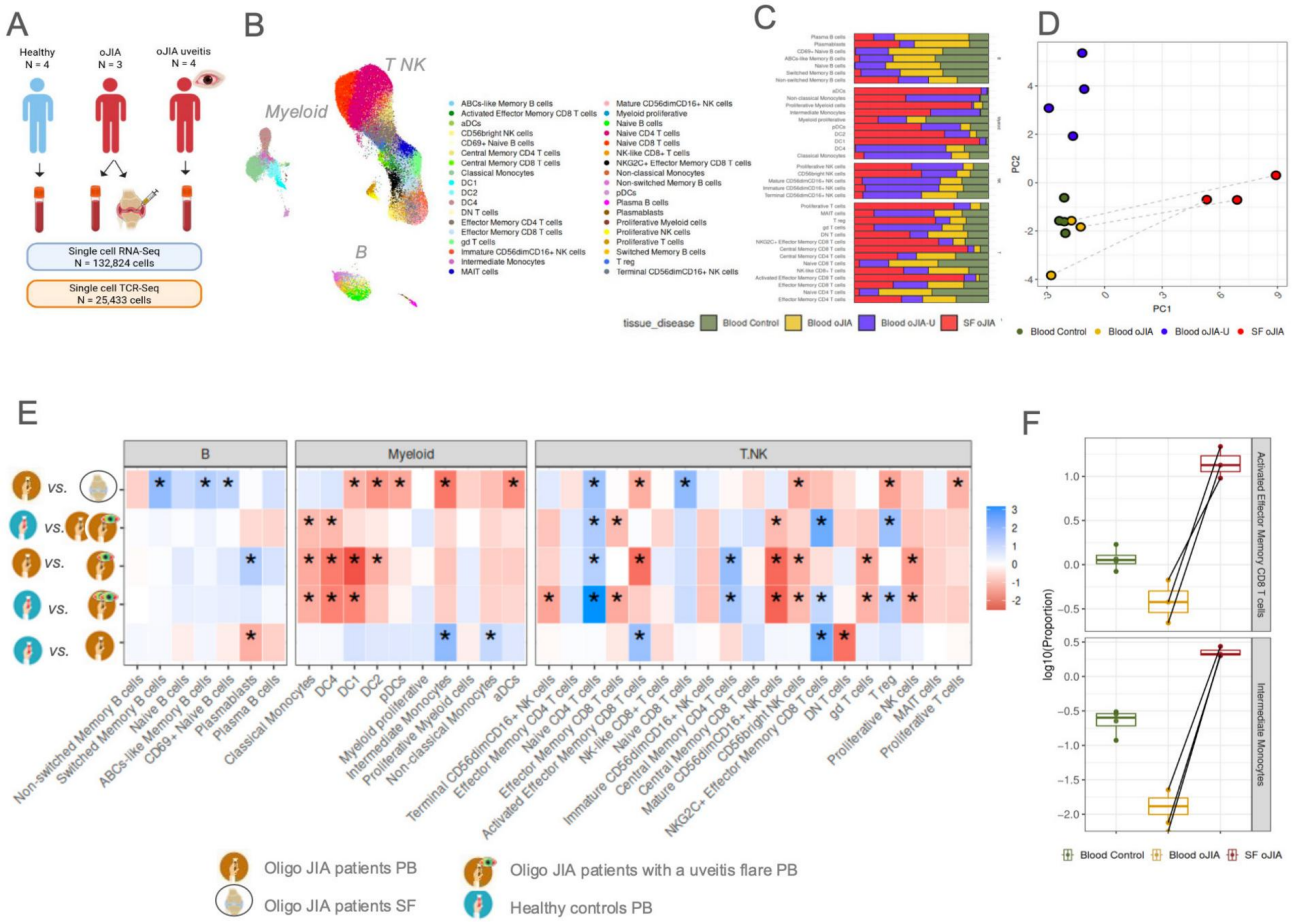
Cellular landscape of oligo JIA and oligo JIA uveitis. (**A**) Collection of PBMCs and SF samples from oligo JIA donors and healthy controls. (**B**) UMAP of PBMCs profiled with scRNA-Seq from blood and synovial samples of 11 donors. Cells of each compartment were independently clustered and annotated into 36 different categories. (**C**) Cell composition across all the groups. (**D**) PCA of cell composition from all samples. Samples from the same individual are linked with dotted lines. (**E**) Differences in cell proportions between paired conditions. Colour gradient represents *t* test results (*t* test statistic *x* − log10 (*P*-value)). Positive values indicate an increase of cell population in the first condition of each pair represented in the *y*-axis. Significant results (*P*-value <0.1 was used as an exploratory discovery threshold given the small sample size of the discovery cohort) are depicted with an asterisk. (**F**) Cell types showing the migration pattern, i.e. significantly more abundant in the synovial fluid of oligo JIA samples vs blood (paired test) and less abundant in oligo JIA blood compared with blood from healthy controls

Matched PBMCs and SF samples from three treatment-naïve children with new-onset oligoarticular JIA were analysed. To study uveitis immunopathology, PBMCs from four oligo JIA patients during uveitis flare and four age- and sex-matched HCs were included. The oligo JIA discovery cohort reflected the classical clinical phenotype of the disease. Patients with new-onset oligo JIA were young (median age 5.3 years), predominantly female (66.7%) and uniformly ANA-positive (100%), with active arthritis and elevated inflammatory markers at diagnosis (median ESR 67.3 mm/h; median CRP 3.29 mg/dl). In contrast, patients sampled during uveitis flare were older (median age 13.8 years), also predominantly female (100%) and ANA-positive (100%), had long-standing disease (median JIA duration 11 years), inactive arthritis at sampling and low systemic inflammatory markers, reflecting isolated ocular activity. These characteristics are consistent with the known demographic and clinical features of oligoarticular JIA and JIA-associated uveitis. Clinical details are summarized in Supplementary Table S1; Fig. 1A.

After quality control, scRNA-seq identified 132 824 immune cells, classified into 36 immune cell types by unsupervised clustering and manual annotation (Fig. 1B; Supplementary Figs. S1–S7). SF samples displayed a markedly distinct immune landscape compared with matched PBMCs (Fig. 1C and D), dominated by activated effector memory CD8+ T cells (14%), followed by effector memory CD4+ T cells (8.8%) and CD8+ TEM cells (7.9%).

Differential abundance analysis compared (1) SF vs matched PBMCs in oligo JIA, (2) PBMCs from oligo JIA vs controls and (3) PBMCs from oligo JIA with uveitis vs controls (Fig. 1E). In blood, oligo JIA patients showed reduced activated CD8+ TEM cells and intermediate monocytes compared with the controls (Fig. 1E and F), consistent with recruitment from the circulation within the inflamed synovial compartment.

PBMCs from oligo JIA patients with uveitis showed a globally distinct immune composition compared with oligo JIA without uveitis and the controls (Fig. 1C and D). Although naïve CD4+ T cells were the most abundant population, their frequency was comparable to the controls. Uveitis was associated with expansion of classical monocytes, DC4, DC1 and multiple NK subsets, including proliferative NK cells (Fig. 1E), indicating a stronger involvement of innate immunity.

### Differential gene expression in oligo JIA and oligo JIA uveitis flare and HCs

**Figure 2:**
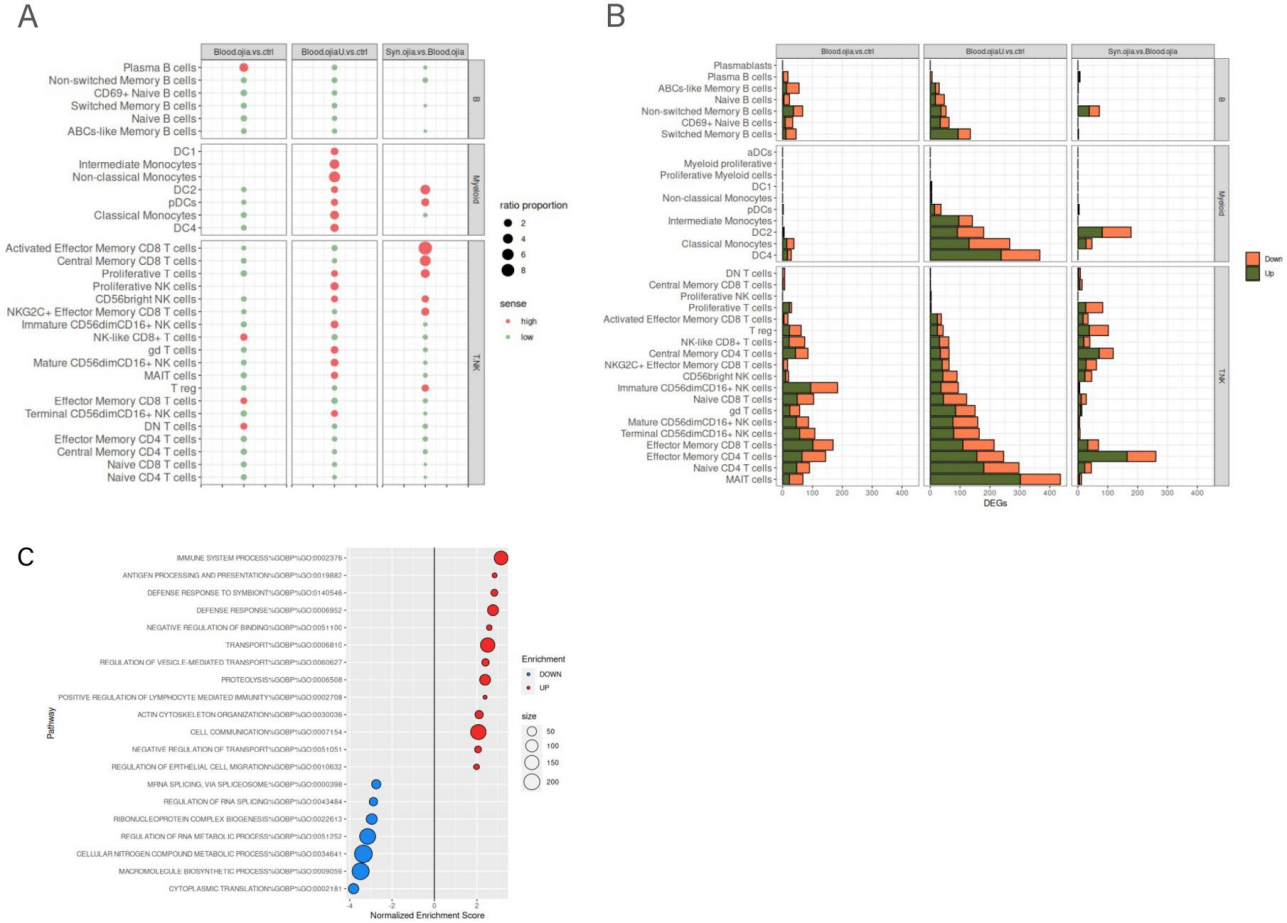
Transcriptional alterations observed in blood and synovial immune cells. (**A**) Ratio of cell type proportions between each pair of conditions (vertical panels: blood oligo JIA vs blood controls, blood oligo JIA uveitis vs blood controls and synovial oligo JIA vs blood oligo JIA). Per each comparison, only ratios (dots) for cell types showing at least one differentially expressed gene (DEG) are represented. (**B**) Number of DEGs per cell type found in each comparison. Upregulated genes are depicted in green, and downregulated genes in orange. (**C**) GSEA in MAIT cells in blood comparing oligo JIA uveitis vs controls

Differential gene expression (DEG) analysis was performed for three comparisons: SF vs matched PBMCs in oligo JIA, PBMCs from oligo JIA vs controls and PBMCs from oligo JIA with uveitis vs controls. Cell expansion and contraction were analysed in cell types with ≥1 DEG (Fig. 2A). Activated CD8+ TEM cells showed the greatest expansion in SF compared with blood, reinforcing their pathogenic relevance.

Effector memory CD4+ T cells exhibited the largest transcriptional reprogramming between blood and SF (261 DEGs; Fig. 2B; Supplementary Table S4), followed by DC2 (178 DEGs) and central memory CD4+ T cells (118 DEGs). In SF, effector memory CD4+ T cells displayed a pro-inflammatory and cytotoxic profile, with strong upregulation of CCL5 (adjusted *P* = 1.24e−54), GZMA (adjusted *P* = 4.12e−43), GNLY (adjusted *P* = 3.4e−11) and HLA-DRB1. Proliferative T cells showed increased IFNG expression (11% in SF vs 1.1% in PB; *P* = 0.002), consistent with a Th1-skewed response in oligo JIA [5]. TOX expression was enriched in proliferative and regulatory T cells in SF, corroborating previously described exhaustion programs [6].

In PBMCs from oligo JIA patients vs controls, immature CD56dimCD16+ NK cells showed the largest transcriptional changes (184 DEGs), followed by effector memory CD8+ and CD4+ T cells, indicating coordinated activation of adaptive and innate immunity. Effector memory CD8+ T cells exhibited increased CX3CR1 and TRBV7-9 expression, suggesting enhanced migratory capacity and antigen-driven expansion.

In uveitis, MAIT cells showed the strongest transcriptional activation (436 DEGs; Fig. 2B), followed by DC4, naïve CD4+ and effector memory CD4+ T cells. MAIT cells upregulated IFN-stimulated genes (IFI44L, ISG15), PRF1 and CORO1A, consistent with cytotoxic activation. Gene set enrichment analysis revealed strong activation of immune and antigen presentation pathways (Fig. 2C; Supplementary Table S5), supporting a role for MAIT cells in uveitis pathogenesis.

Comparison between oligo JIA with and without uveitis revealed no uniquely enriched immune pathways, suggesting that uveitis represents an amplification rather than a qualitative shift of shared inflammatory programs.

### Clonal expansion of effector memory CD8 T cells in oligo JIA

**Figure 3:**
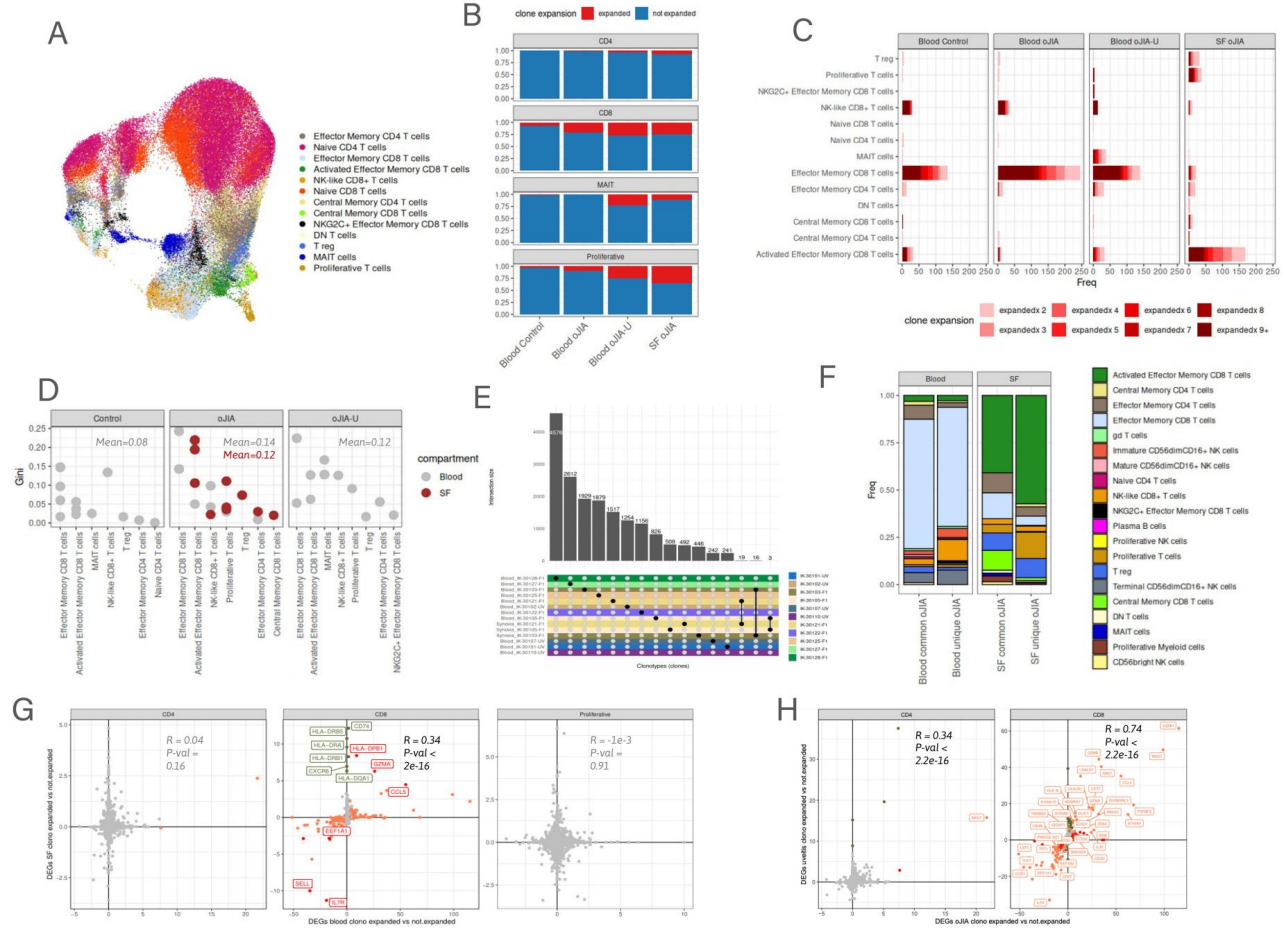
Analysis of clonal expansion in oJIA using TCR-seq. (**A**) UMAP of scRNA-seq data from TCR-sequenced cells. (**B**) Fraction of expanded and not expanded clones in CD4 T, CD8 T, MAIT and proliferative T cells in each condition. (**C**) Absolute number of expanded clones categorized by expansion level, grouped by cell type. (**D**) Gini coefficients estimated on clones from different cell types, conditions and tissue. Each dot represents a different sample. (**E**) Absolute number of clones identified in each sample and donor. Shared clones are found only in paired samples from the same donor. (**F**) Distribution of expanded clones found uniquely in the blood of oJIA donors (Blood_unique_oJIA), uniquely in the SF of oJIA donors (Synovia_unique_oJIA) or commonly found in both tissues (Blood_common_oJIA and Synovia_common_oJIA). (**G**) Foldchange and significance comparison of DEGs computed in expanded vs not expanded clones in circulating PBMCs (*x*-axis) with DEGs computed in expanded vs not expanded clones in SF from oJIA (*y*-axis). (**H**) Foldchange and significance comparison of DEGs computed in expanded vs not expanded clones in circulating PBMCs from oJIA donors (*x*-axis) with DEGs computed in expanded vs not expanded clones in PBMCs from oJIA with uveitis (*y*-axis). Genes with a significant different expression level in both comparisons are coloured in red. DEGs only in blood are in orange, and DEGs only in SF are in green. Spearman correlation test estimates and *P*-values are depicted in each plot

Effector memory CD8+ T cells were the most enriched population in SF and depleted in blood, indicating active recruitment. TCR sequencing was performed to assess clonal dynamics. After stringent filtering (≥10% TCR coverage; ≥30 cells) [10], 25 433 T cells were analysed (Fig. 3A and B). CD8+ T cells showed significant clonal expansion in SF, particularly proliferative CD8+ clones (*P* = 7.2e−6), whereas CD4+ T cells showed minimal expansion.

In blood from oligo JIA patients with uveitis, clonally expanded MAIT cells were identified (*P* = 3.7e−10; Fig. 3C), consistent with transcriptional activation observed above. No public CD8+ clonotypes were shared across donors (Fig. 3E), but shared clonotypes between blood and SF within individuals were frequent (38 shared clones; *P* = 7.7e−58; Fig. 3F).

Expanded CD8+ T cell clonotypes in SF showed 14 DEGs compared with non-expanded clones (Supplementary Table S6), including HLA-DRB5, CXCR6 and HLA-DRA, genes associated with antigen presentation and tissue residency [13] (Fig. 3G). No transcriptional differences were detected in expanded CD4+, proliferative T or MAIT clones.

Expanded T cell clones in oligo JIA blood vs controls showed 182 DEGs, predominantly in CD8+ T cells. Gene expression profiles of expanded CD8+ T cells in blood and SF were positively correlated (*R* = 0.34, *P* < 2e−16), with shared upregulation of CCL5, GZMA and HLA-DPB1 (Supplementary Table S5), suggesting peripheral priming before joint infiltration.

Comparative analysis of expanded clones in oligo JIA with and without uveitis revealed shared cytotoxic programs but greater transcriptional intensity in uveitis, including upregulation of GNLY, GZMK, GZMH and CCL5 (Fig. 3H), indicating amplified effector function associated with ocular involvement.

### Validation of changes in immune populations in oligo JIA using mass cytometry (CyTOF)

**Figure 4:**
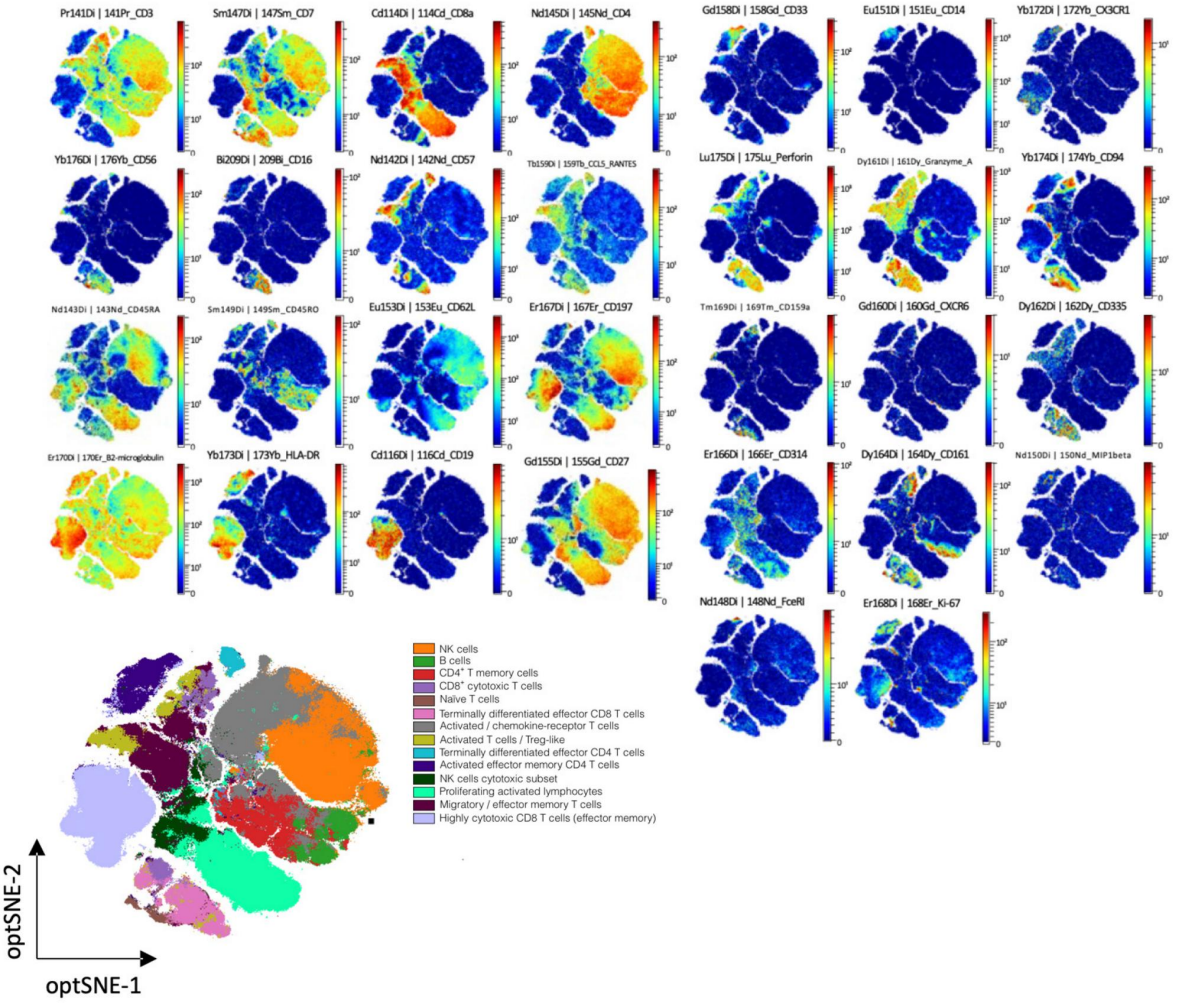
Computational clustering of the validation dataset using CyTOF. PBMCs of oligo JIA patients and healthy controls were analysed using the defined panel of markers and clustering was performed using the FlowSOM algorithm. A total of 14 clusters were identified representing the main circulating immune types. The expression of each marker is displayed using a colour heat scale going from blue (low expression) to red (high expression) on the optSNE plot

Key immune alterations were validated by CyTOF using a 31-antibody panel (Supplementary Table S3) in an independent cohort. OptSNE and FlowSOM identified 14 clusters (Fig. 4).

Peripheral blood analysis confirmed reduced activated CD8+ TEM cells in oligo JIA compared with the controls (7.7% vs 15.2%; *P* = 0.046), consistent with recruitment from the circulation to inflamed synovial compartment. Activated CD8+ TEM cells showed increased intracellular CCL5 expression (27.5% vs 15.4%; *P* = 0.0021), and expansion of a GZMA+ CD8+ subset (59.8% vs 23.6%; *P* = 0.0055).

CyTOF also validated NK cell alterations, including expansion of CD161+ NK cells (37.68% vs 21.57%; *P* = 0.002) and proliferative Ki-67+ NK subsets (4.16% vs 0.52%; *P* = 0.0021), consistent with a pro-inflammatory, tissue-adapted innate response.

Together, CyTOF data confirm the expansion and activation of effector CD8+ T cells and NK subsets, validating the transcriptomic findings and supporting a pathogenic immune landscape in oligoarticular JIA.

## Discussion

In this study, we provide an integrated single-cell transcriptomic, T cell receptor repertoire and mass cytometry characterization of the immune landscape in oligo JIA and its ocular complication, uveitis. By analysing paired SF and peripheral blood samples from treatment-naïve patients, together with blood samples from uveitis cases and matched HCs, we identify distinct cellular and molecular signatures underlying local joint inflammation and systemic immune dysregulation.

Single-cell analysis revealed marked immune compartmentalization in oligo JIA. SF was enriched in activated effector memory CD8^+^ and CD4^+^ T cells, intermediate monocytes, regulatory and proliferative T cells, with depletion of naïve T and B cells compared with blood. Notably, clonally expanded effector memory CD8^+^ T cells were highly enriched in SF but detectable in blood, sharing transcriptional programs dominated by CCL5, GZMA and GNLY. Their reduced frequency in circulation supports active recruitment to the inflamed joint, where these antigen-experienced cytotoxic T cells likely contribute directly to tissue damage.

CD8^+^ T cell expansion in oligo JIA was associated with a coherent transcriptional program involving chemotaxis, cytotoxicity and antigen presentation, indicating both quantitative expansion and qualitative functional specialization. In patients with uveitis, peripheral blood immune profiles differed markedly from both HCs and oligo JIA patients without uveitis.

Systemic immune activation signatures during uveitis flare, including MAIT cell expansion and IFN-stimulated gene expression, parallel observations described in non-JIA autoimmune uveitis and other inflammatory ocular disorders. This suggests that uveitis-associated immune activation may partially reflect shared mucosal–systemic inflammatory axes rather than arthritis-specific mechanisms. Nevertheless, the identification of clonally expanded MAIT populations in JIA-associated uveitis highlights a potentially distinct cellular contributor in this paediatric context.

### Immune alterations in oligo JIA and uveitis

Our findings are consistent with previous single-cell studies reporting Th1 skewing, memory T cell predominance and exhaustion programs in synovial T cells, although these studies were limited by small sample size or use of adult controls [5, 6, 14]. Despite these limitations, we validate and extend these observations in a paediatric, treatment-naïve cohort with HCs.

In oligo JIA-associated uveitis, peripheral blood showed expansion of classical monocytes, DC1/DC4 subsets and proliferative NK cells. Monocyte involvement in non-infectious uveitis has been previously reported [15], while dendritic and NK cell contributions have been implicated in other inflammatory uveitic diseases such as Behçet’s disease and Vogt–Koyanagi–Harada syndrome [16]. Our results suggest that similar innate immune mechanisms may operate in JIA-associated uveitis.

### Effector memory CD8^+^ T cells as drivers of joint inflammation

Effector memory CD8^+^ T cells emerged as central pathogenic players in oligo JIA. These cells displayed clonal expansion, cytotoxic and tissue-resident features and shared transcriptional profiles across blood and synovial compartments, consistent with systemic antigen-driven activation followed by local amplification. CCL5 overexpression in synovial CD8^+^ T cells has been previously described [17], and similar cytotoxic CD8^+^ T cell expansions have been reported in PsA, where tissue-resident or activated CD8^+^ T cells with cytotoxic transcriptional signatures have been implicated in local inflammation [18]. Thus, elements of the transcriptional program identified here likely represent conserved inflammatory pathways rather than mechanisms unique to oligoarticular JIA. However, our study extends these observations to early, treatment-naïve paediatric disease and demonstrates marked clonal expansion within the inflamed joint, supporting antigen-driven responses at disease onset.

Given the clinical and therapeutic overlap between PsA and oligo JIA, including the efficacy of IL-17A blockade in juvenile PsA and enthesitis-related arthritis [19], our findings suggest overlapping immunopathogenic mechanisms. Although oligo JIA has not been directly studied in IL-17-targeted trials, the shared expansion of cytotoxic CD8^+^ T cells raises the possibility that selected patients may benefit from similar therapeutic strategies. GZMA, consistently expressed by pathogenic CD8^+^ T cells in SF, further supports their role in mediating local inflammation and joint damage [5, 20].

### Systemic immune activation and MAIT cell involvement

Peripheral blood transcriptomics revealed systemic immune activation in oligo JIA, with altered effector memory CD4^+^ and CD8^+^ T cells. Upregulation of CX3CR1 and TRBV7-9 in CD8^+^ T cells supports enhanced tissue homing and antigen-driven clonal selection [21–23]. Similar cytotoxic effector programs have been described in adult inflammatory arthritis, suggesting that these transcriptional signatures may reflect shared inflammatory pathways rather than disease-specific mechanisms. These findings suggest that expanded CD8^+^ effector memory T cells may target shared self-antigens, warranting further repertoire analysis.

In uveitis, MAIT cells showed pronounced transcriptional activation, including IFN-stimulated genes and cytotoxic mediators. MAIT cells are innate-like T lymphocytes enriched at mucosal and barrier sites and can be activated through both TCR-dependent recognition of microbial riboflavin metabolites presented by MR1 and cytokine-mediated pathways independent of antigen specificity.

In particular, IL-12, IL-18 and type I IFNs can induce rapid MAIT activation and acquisition of cytotoxic programs, consistent with the IFN-stimulated signatures observed in our cohort [24]. Prior studies have linked MAIT cells to IL-17/IFN-γ-driven inflammation in autoimmune uveitis and have proposed a gut-systemic-eye axis connecting mucosal barrier perturbation to ocular inflammation [25–28], supporting their potential pathogenic role in JIA-associated uveitis. These interpretations remain hypothesis-generating and warrant further investigation.

### Clonal expansion links systemic and local inflammation

Single-cell TCR sequencing demonstrated robust clonal expansion of effector memory CD8^+^ T cells in SF, with shared clonotypes and overlapping transcriptional programs between blood and joint, particularly involving CCL5, GZMA and HLA-DPB1. The absence of comparable expansion in conventional CD4^+^ T cells highlights a predominant effector role for CD8^+^ and MAIT cells in early oligo JIA [29], implicating antigen-specific CD8^+^ T cell responses as key drivers of joint and potentially ocular inflammation.

### CyTOF validation and clinical implications

CyTOF analysis in an independent cohort confirmed the reduction of activated CD8^+^ effector memory T cells in blood, increased expression of GZMA and CCL5 and expansion of proliferative NK cell subsets. These findings align with reports of aberrant type 1 cytokine signalling and IFN-γ/STAT1 hypersensitivity in JIA [30]. Concordant transcriptional and protein-level signatures highlight CCL5, GZMA, Ki-67 and CD161 as candidate biomarkers and mechanistic targets. Therapeutically, disruption of the CCL5–CCR5 axis may represent a strategy to limit pathogenic CD8^+^ T cell recruitment in patients with a dominant cytotoxic immune phenotype [31].

### Limitations and future directions

Limitations include the small discovery cohort and treatment exposure in uveitis patients, which may have influenced circulating immune profiles. Nevertheless, persistent inflammatory signatures despite therapy suggest disease-specific mechanisms. Comparison of blood and synovial compartments requires caution, as peripheral blood represents a small fraction of the systemic immune pool, whereas SF sampling captures a larger proportion of the local inflammatory niche. Metrics of clonal inequality, such as the Gini coefficient, may therefore partially reflect compartment size and sampling depth. Depletion in blood and enrichment in SF are consistent with recruitment but do not exclude local proliferation or tissue retention.

In addition, SF samples required short *ex vivo* processing steps, including hyaluronidase treatment to reduce viscosity, which could theoretically induce transient transcriptional perturbations. Although samples were processed rapidly under standardized conditions and uniformly handled across all patients, we cannot fully exclude minor *ex vivo* effects on gene expression profiles.

Larger, longitudinal studies are needed to define disease trajectories, antigen specificity of pathogenic clones and treatment responses. Antigen discovery approaches such as peptide–MHC multimer staining may clarify CD8^+^ T cell targets [32]. Age differences between the groups represent a limitation, as immune composition varies across childhood. Future studies should include tighter age matching and age-adjusted analyses in larger cohorts. Future studies incorporating additional comparator groups—including new-onset oligo JIA with concurrent uveitis, treated inactive uveitis, other JIA subtypes and non-JIA uveitis—will be essential to define disease-specific vs shared inflammatory signatures.

In addition, *in silico* validation using publicly available single-cell datasets represents a feasible next step to test the reproducibility of the signatures identified here.

Finally, future integration of scRNA-seq, scTCR-seq, CyTOF and spatial transcriptomics will be essential to define cell–cell interactions and tissue niches driving joint inflammation in oligo JIA.

## Supplementary Material

keag211_Supplementary_Data

## Data Availability

The single-cell RNA sequencing and T cell receptor sequencing datasets generated during this study have been deposited in the NCBI repository (SUB15430472 [PRJNA1294527]). Additional data supporting the findings of this study, including processed data and metadata, are available from the corresponding author upon reasonable request.
