# Supplementary material for: Single-cell profiling reveals CCL5Hi GZMAHi effector memory CD8 T cell association to oligoarticular JIA
Source: Rheumatology (Oxford). 2026 Apr 27;65(6):keag211. doi: 10.1093/rheumatology/keag211 (PMC13278834; doi:10.1093/rheumatology/keag211)
Supplement: keag211_Supplementary_Data [file keag211_supplementary_data.zip › rhe-25-3245-File004.docx]

**SUPPLEMENTARY DATA**

**Supplementary Table S1. Baseline characteristics of the study population**

| Variable | Oligo JIA debut  (N= 3) | Uveitis flare  (N= 4) | Healthy controls  (N= 4) | *P*-value |
| --- | --- | --- | --- | --- |
| Age | 5.33 [3.50-6.50] | 13.75 [13.50-14.25] | 11.25 [9.75-15.00] | 0.13 |
| Gender | F   (2/3, 66.7%) | F   (4/4, 100.0%) | F   (3/4, 75.0%) | 0.45 |
| Years disease duration | 0 | 11.00 [9.50-12.50] | - | 0.04 |
| Years uveitis duration | - | 7.25 [6.00-8.25] | - | - |
| ANA | P   (3/3, 100.0%) | P   (4/4, 100.0%) | - | 0.04 |
| CRP (mg/dl) | 3.29 [0.34-4.80] | 0.04 [0.02-0.06] | - | 0.05 |
| ESR (mm/h) | 67.33 [44.50-86.50] | 12.75 [4.50-19.25] | - | 0.06 |
| Active joints | Yes   (3/3, 100.0%) | No   (4/4, 100.0%) | - | 0.01 |
| JADAS27 | 15.07 [11.95-16.65] | 2.40 [0.00-4.70] | - | 0.05 |
| SUN cells | - | 0.5+   (3/4, 75.0%) | - | - |
| SUN flare | - | 0   (3/4, 75.0%) | - | - |
| bDMARD | - | ADA   (4/4, 100.0%) | - | - |
| cDMARD | - | MTX   (3/4, 75.0%) | - | - |

Data are presented as median [IQR] for continuous variables and n/N (%) for categorical variables. Kruskal–Wallis was applied when all three groups (including Healthy controls) had available data (e.g., Age). Mann–Whitney U test (pairwise Oligo JIA vs Uveitis) was used when Healthy controls had no available data (e.g., Years JIA, CRP, ESR).
Categorical variables were summarized as counts (%) and compared using Chi-square tests with corresponding p-values.

ADA: adalimumab; ANA: antinuclear antibodies; bDMARDs: biologic disease-modifying antirheumatic drugs; cDMARDs: conventional disease-modifying antirheumatic drugs; CRP: c-reactive protein (mg/dl); ESR: erythrocyte sedimentation rate (mm/hour); F: female; Gen: Gender; JADAS27: composite juvenile idiopathic disease activity measure, 27 joints evaluated; LFN: leflunomide; M: male; MTX: methotrexate; P: positive; SUN: Standardization of Uveitis Nomenclature; UV: uveitis-related oligoarticular juvenile idiopathic arthritis.

**Supplementary Table S2. Patient demographics from the validation cohort analyzed with CyTOF.**

| Variable | Oligo JIA  (N= 13) | Uveitis flare  (N= 8) | Healthy controls  (N= 6) | *P*-value |
| --- | --- | --- | --- | --- |
| Age | 8.15 [4.00-12.00] | 12.00 [10.25-14.25] | 7.00 [3.25-8.75] | 0.08 |
| Gender | F:9 (69.2%) | F:8 (100.0%) | F:4 (66.7%) | 0.19 |
| Years disease duration | 1.46 [0.00-0.00] | 8.75 [6.25-12.00] | - | 0.01 |
| Years uveitis duration | - | 5.25 [2.75-7.00] | - | - |
| CRP (mg/dl) | 1.03 [0.05-0.30] | 0.12 [0.03-0.10] | - | 0.17 |
| ESR (mm/h) | 28.46 [10.00-35.00] | 15.50 [12.25-18.25] | - | 0.17 |
| JADAS27 | 6.96 [3.00-11.50] | 1.20 [0.00-1.15] | - | 0.01 |
| ANA | P   (13/13, 100.0%) | P   (8/8, 100.0%) | - | 0.41 |
| Active_joints | Yes   (13/13, 100.0%) | No   (8/8, 100.0%) | - | 0.03 |
| cDMARD | MTX:4 (80.0%);  LFN:1 (20.0%) | MTX:5 (71.4%);  LFN:2 (28.6%) | - | 1.00 |
| bDMARD | - | ADA:6 (100.0%) | - | - |

Data are presented as median [IQR] for continuous variables and n/N (%) for categorical variables. p-values were calculated using Kruskal–Wallis test (continuous variables) or chi-squared test (categorical variables).

ADA: adalimumab; ANA: antinuclear antibodies; bDMARDs: biologic disease-modifying antirheumatic drugs; cDMARDs: conventional disease-modifying antirheumatic drugs; CRP: C-reactive protein (mg/dl); ESR: erythrocyte sedimentation rate (mm/hour); F: female; HC: healthy control; JADAS27: composite juvenile idiopathic disease activity measure, 27 joints evaluated; LFN: leflunomide; M: male; MTX: methotrexate; cDMARD: oligoarticular juvenile idiopathic arthritis under conventional disease-modifying antirheumatic drugs; P: positive.

**Supplementary Table S3. Antibody panel designed for the validation study of immune cell association to oligo JIA using CyTOF.**

| Specificity | Isotope | Clone | Surface/Intracellular | Manufacturer |
| --- | --- | --- | --- | --- |
| CD45 | 89Y | HI30 | Surface | Standard BioTools |
| CD8a* | 114Cd | RPA-T8 | Surface | BioLegend |
| CD19* | 116Cd | HIB19 | Surface | BioLegend |
| CD3 | 141Pr | UCHT1 | Surface | Standard BioTools |
| CD57 | 142Nd | HCD57 | Surface | Standard BioTools |
| CD45RA | 143Nd | HI100 | Surface | Standard BioTools |
| CD4 | 145Nd | RPA-T4 | Surface | Standard BioTools |
| CD7 | 147Sm | CD7-6B7 | Surface | Standard BioTools |
| FceRI* | 148Nd | AER-37 (CRA-1) | Surface | BioLegend |
| CD45RO | 149Sm | UCHL1 | Surface | Standard BioTools |
| MIP-1b (CCL4) | 150Nd | D21-1351 | Intracellular | Standard BioTools |
| CD14 | 151Eu | M5E2 | Surface | Standard BioTools |
| CD62L | 153Eu | DREG-56 | Surface | Standard BioTools |
| CD27 | 155Gd | L128 | Surface | Standard BioTools |
| CD33 | 158Gd | WM53 | Surface | Standard BioTools |
| CCL5 (RANTES)* | 159Tb | VL1 | Intracellular | BioLegend |
| CXCR6 (CD186) | 160Gd | K041E5 | Surface | Standard BioTools |
| Granzyme A* | 161Dy | CB9 | Intracellular | BioLegend |
| NKp46 (CD335) | 162Dy | BAB281 | Surface | Standard BioTools |
| CD161 (KLRB1) | 164Dy | HP-3G10 | Surface | Standard BioTools |
| NKG2D (CD314) | 166Er | ON72 | Surface | Standard BioTools |
| CCR7 (CD197) | 167Er | G043H7 | Surface | Standard BioTools |
| Ki67 | 168Er | B56 | Intracellular | Standard BioTools |
| NKG2A (CD159a) | 169Tm | Z199 | Surface | Standard BioTools |
| b2 microglobulin | 170Er | 2M2 | Surface | Standard BioTools |
| CX3CR1* | 172Yb | 2A9-1 | Surface | BioLegend |
| HLA-DR | 173Yb | L243 | Surface | Standard BioTools |
| CD94 | 174Yb | HP-3D9 | Surface | Standard BioTools |
| Perforin | 175Lu | B-D48 | Intracellular | Standard BioTools |
| CD56 (NCAM) | 176Yb | NCAM16.2 | Surface | Standard BioTools |
| CD16 | 209Bi | 3G8 | Surface | Standard BioTools |

*Denotes antibodies that were conjugated with Maxpar MCP9 and X8 Labelling kits.

**Supplementary Table S4:** Differentially expressed genes between each comparison.

**Supplementary Table S5:** Differentially expressed genes between exposed and non-exposed JIA patients in peripheral blood.

**Supplementary Table S6:** Differentially expressed genes between exposed and non-exposed JIA patients in peripheral blood.

**Supplementary Figure S1.** **Cell annotation of PBMCs.** (A) Louvain clustering of PBMCs at resolution 0.2. (B) Annotation of major immune compartments based on the expression of key markers displayed in (C).

**Supplementary Figure S2.** **Cell annotation of myeloid cells.** (A) Louvain clustering of myeloid cells at resolution 0.25. (B) Annotation of myeloid cells based on the expression of key markers displayed in (C).

**Supplementary Figure S3.** **Cell annotation of B cells.** (A) Louvain clustering of B cells at resolution 0.5. (B) Annotation of B cells based on the expression of key markers displayed in (C).

**Supplementary Figure S4.** **Manual cell annotation of T and NK cells.**(A) Louvain clustering of T and NK cells at resolution 1.3. (B) Annotation of T and NK cells based on the expression of key markers displayed in (C).

**Supplementary Figure S5.** **Subclustering and annotation of NK cells.** (A) Louvain clustering of NK cells at resolution 1.3. (B) Annotation of NK cells based on the expression of key markers displayed in (C).

**Supplementary Figure S6.** **Subclustering and annotation of CD4+, CD8+, and non conventional T cells.** (A) Louvain clustering of T cells, excluding Naive CD4+ and Naive CD8+ T cells, at resolution 1.5. (B) Expression of gene markers for MAIT (TRAV1-2) and gd-T cells (TRGV9, and TRDC). (C) Different CD4 T cell subtypes annotated. Tregs are identified based on the expression of genes depicted in (D), CD4 memory and non conventional CD4 T cells are annotated based on the expression of genes represented in (E).

**Supplementary Figure S7.** **Subclustering and annotation of CD8+ T cells.**(A) Subclustering of non-Naive CD8 T cells (see Supplementary Figure 4). Different CD8 T cell subtypes are annotated according to the expression of the depicted genes. (B) Different CD8 T cell subtypes annotated.
